# Retrospective analysis of operative time and time to discharge for laparoscopic vs robotic approaches to appendectomy and cholecystectomy

**DOI:** 10.1007/s11701-023-01632-9

**Published:** 2023-06-04

**Authors:** Ahmad Oussama Rifai, Emily M. Rembetski, Larry Collins Stutts, Zachary D. Mazurek, Jenifer L. Yeh, Kareem Rifai, Ryan A. Bear, Alexander J. Maquiera, David J. Rydell

**Affiliations:** 1 The Education and Research Department, The Virtual Nephrologist, INC, PO Box 1750, Lynn Haven, FL 32444-5950 USA; 2 ACOM, Research Department, Alabama College of Osteopathic Medicine, 445 Health Sciences Boulevard, Dothan, AL 36303 USA; 3 HCA Florida Trinity Hospital, 9330 SR 54 E, Trinity, FL 34655 USA; 4 Envision Physician Services, HCA Florida Gulf Coast Hospital, 449 west 23rd stree, Panama City, FL 32405 USA

**Keywords:** Robotic-assisted appendectomy, Robotic-assisted cholecystectomy, Laparoscopic, Intraoperative time, Time to discharge

## Abstract

Robotic-assisted appendectomies and cholecystectomies are believed to increase cost compared to the gold standard laparoscopic approach. Two equally qualified surgeons performed both approaches over 2 years to evaluate intraoperative duration, time to discharge, conversion to open procedure, and readmission within 30 days. 110 laparoscopic, 81 robotic-assisted appendectomies; and 105 laparoscopic and 165 robotic-assisted cholecystectomies were performed. Intraoperative time; laparoscopic appendectomy was 1.402 vs 1.3615 h for robotic-assisted (*P *value = 0.304); laparoscopic cholecystectomy was 1.692 vs 1.634 h for robotic-assisted (*P* value = 0.196). Time to discharge, was 38.26 for laparoscopic vs 28.349 h for robotic-assisted appendectomy (*P* value = 0.010), and 35.95 for laparoscopic vs 28.46 h for robotic-assisted cholecystectomy (*P* value = 0.002). Intraoperative conversion to open; only laparoscopic procedures were converted, one appendectomy and nine cholecystectomies. None in the robotic-assisted procedures. Readmissions, none in the appendectomy group and three in the cholecystectomy group. One laparoscopic and two robotic-assisted cholecystectomy patients were readmitted. Intraoperative times for robotic appendectomy and cholecystectomy were not longer than laparoscopic approach. Robotic approach shortened the time to discharge and the likelihood for conversion to open procedure.

## Introduction

The muscle splitting approach for appendectomy was introduced by Charles McBurney in 1893 and served as the standard approach for a century, until Kurt Semm performed the first laparoscopic appendectomy 1981 [1]. The laparoscopic appendectomy was suggested as the gold standard approach for the surgical treatment of acute and chronic appendicitis later in 1997 [2]. Supporting this, a review of discharge data for 20% of acute appendicitis patients in the USA (43,757 patients) was conducted by Guller et al.; when compared with the McBurney open appendectomy, the laparoscopic approach was shown to have a shorter length of stay and fewer hospital morbidities [3]. However, it was not until 2016 when the laparoscopic appendectomy was officially established as the gold standard of care by Guerico et al. and others [4].

In 2008, the first robotic appendectomy was described by Akl et al. as an incidental appendectomy during an elective pelvic gynecological surgical procedure [5]. Later in 2013, Stephen Pereira performed the first robotic-assisted appendectomy for acute appendicitis in Hackensack, New Jersey, USA [6].

Currently in the United States, there are 300,000 cases acute appendicitis per year, and this procedure is considered straightforward for new surgical residents to learn proficiently during training. However, utilizing robotic-assisted appendectomy as the default choice and gold standard can be cost prohibitive for hospitals. In addition, there are issues with operating rooms’ (ORs) expenses and availability of supplies, as well as trained staff, and there is a generation of surgeons who have not received formal training for robotics. Thus, in the absence of strong clinical superiority and cost savings, surgical departments have not been able to make robotic-assisted appendectomies to be the gold standard and default approach for acute appendicitis.

Gall bladder disease was traditionally treated by cholecystostomy with drainage, and removal of gallstones until the 1880s, when the first successful open cholecystectomy was performed by German physician Carl Johann August Langenbuch. In 1985, the first laparoscopic cholecystectomy was performed, and in 1992 the national institute of health codified the laparoscopic approach as the standard of care for cholecystectomies [7].

However, robotic cholecystectomies have recently become an alternative to the laparoscopic approach. Despite the benefits of the robotic approach, barriers to obtaining an appropriate level of surgeon workforce and lack of clear clinical superiority have kept robotic cholecystectomy in an alternative position.

Thus, the aim of this study was to evaluate and compare intraoperative duration and post-operative length of stay for the laparoscopic approach vs that of the robotic approach. In addition, we aimed to evaluate the rate of conversion to open procedures as well as the rate of readmissions within 30 days [8–10].

## Methods

The data for this study was collected during a 2-year period between January 1, 2019, and December 31, 2020. Two surgeons rotating on emergency room general surgery call equally in a community Hospital, Gulf Coast Regional Medical Center in Panama City, Florida, USA. As this was a retrospective study, we maintain that each patient received what was medically considered the most appropriate, effective, and safe surgery.

### Selection and randomization of patients

All individuals admitted to the emergency room with a diagnosis of acute appendicitis or acute cholecystitis were evaluated for this study and enrolled only if they underwent an appendectomy or cholecystectomy during the initial hospitalization. A total of 491 patients were included in the study with 30 excluded due to undergoing different procedures (20 operations were begun as open procedures and 10 laparoscopic procedures were converted to open procedures intraoperatively). Thus, the total number of patients analyzed in our study was 461.

A search of electronic medical records for appendectomy or cholecystectomy procedures yielded an additional 63 patients; however, they were all excluded as they were received either elective outpatient procedures or additional surgeries in combination with appendectomy or cholecystectomy.

The study was conceived of and designed 1 year following the end date of the collection period. Thus, the decision to use an open procedure was left solely to the operating surgeon and was based on clinical judgement and patient safety. The decision for intraoperative conversion was also left to the surgeon and was based on technical difficulty and safety.

### Surgeon selection and randomization

Surgeon A and surgeon B were of the same age, had both graduated from US medical school in 1991, and had completed general surgery residencies in 1996. Both were also trained in a university medical center. They were considered to have equal training and clinical experience in open appendectomies and open cholecystectomies, as well as laparoscopic appendectomies and cholecystectomies as an essential part for their general surgery training.

In addition, Surgeon B was considered an expert in Robotic-assisted surgery, having completed 214 Robotic procedures prior to January 1, 2019 [11]. These included 80 robotic cholecystectomies, with the rest general abdominal surgeries, appendectomies, colorectal procedures, Nissen fundoplication, and ventral and inguinal hernia.

In this study, all robotic surgeries were performed by surgeon B, and laparoscopic procedures were performed by either surgeon A or surgeon B. We used a unique randomization of surgeons.

### Primary end points

This study evaluated whether there was a statistically significant difference between the two surgical methods in the two primary end points, OR time and time to discharge.

OR time was defined as the duration of time the patient spent in the OR and was recorded as the time the patient was rolled into the OR until the time the patient was rolled out of the OR, as recorded in the medical record. This was considered a surrogate for OR utilization.

Time to discharge was defined as the duration of time between the time the patient was rolled out of the OR until the time of hospital discharge, as recorded in the medical record. It reflected many clinical factors including post-operative pain and care. Indirectly, it also represented post-operative recovery and potential complications.

These two time periods were viewed as direct surrogates of resource utilization.

### Secondary end points

The first secondary endpoint was intraoperative conversion from laparoscopic or robotic surgery to open procedure. This represented potential additional OR cost and time.

The second secondary endpoint was the rate of readmission within 30 days for all causes, which represented an additional cost for the hospital to absorb.

These two additional secondary endpoints were also evaluated as additional practical factors that may have influenced comparison between the laparoscopic and robotic approaches.

### Hospital background

Post-operative care was provided primarily by hospitalists who were dedicated to the floor patients care and were strongly encouraged to discharge patients, with the surgeon approval, in an expedient fashion.

### Statistical analysis

An independent *t* test was used to determine statistical significance, as the sample sets were not of equal size and the means of the two data sets were not equal. For all analyses, the null hypothesis was that there was no statistical difference between the two data sets [12].

## Results

### Data outliers

**Fig. 1:**
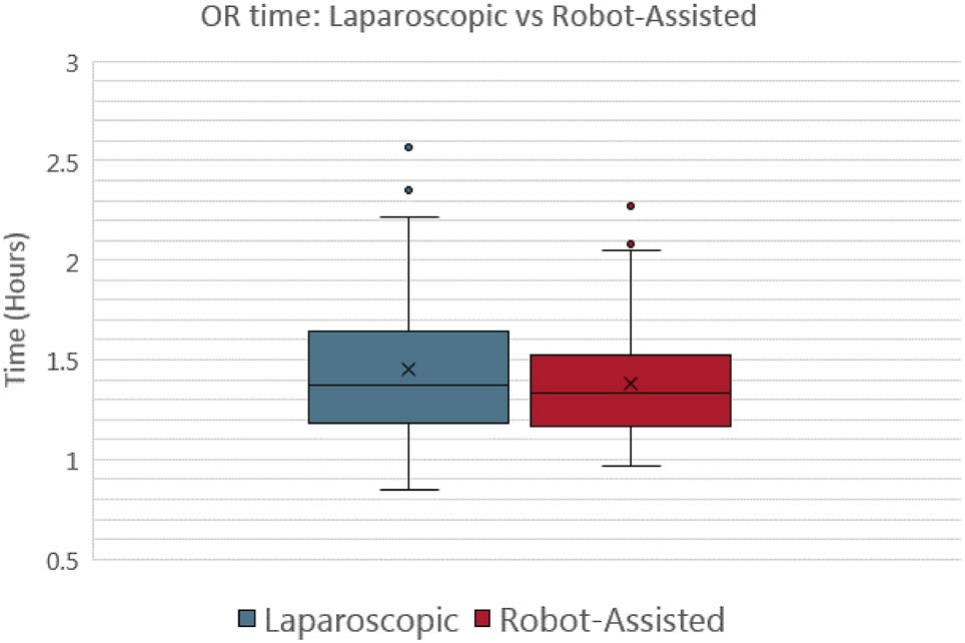
Box-and-whisker plot for appendectomy OR time

**Fig. 2:**
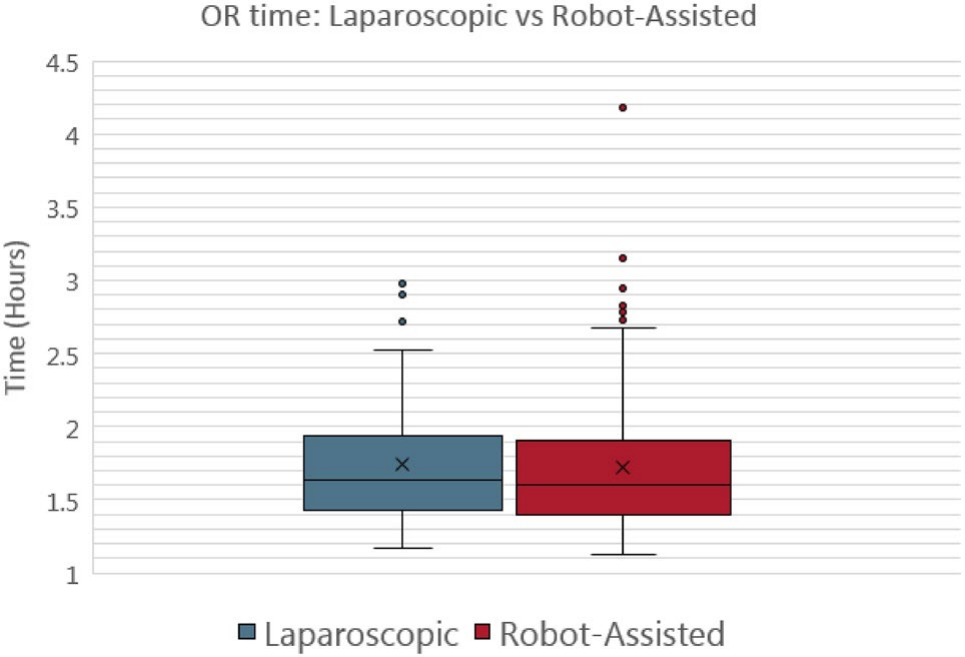
Box-and-whisker plot for cholecystectomy OR time

**Fig. 3:**
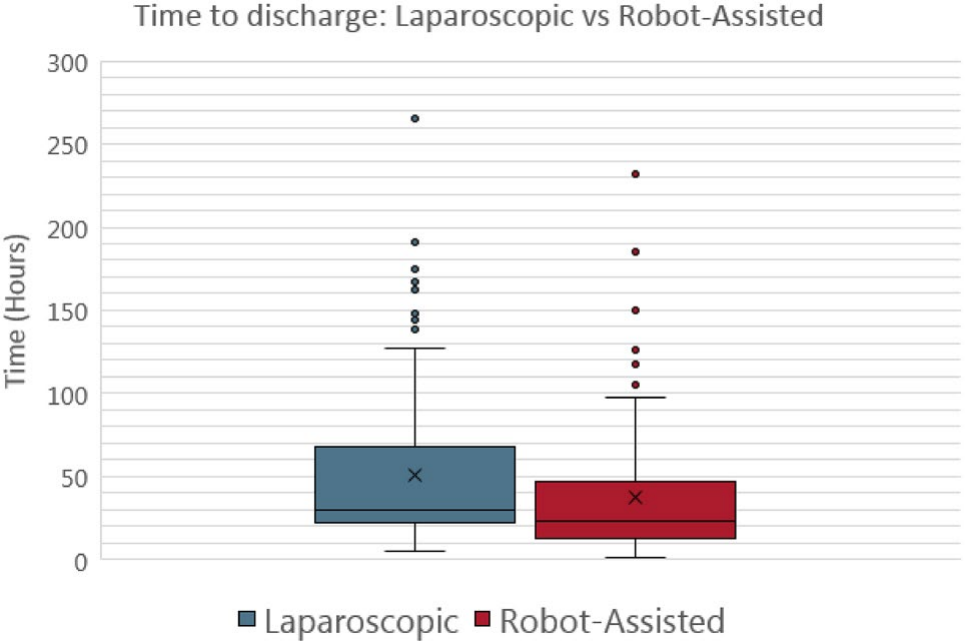
Box-and-whisker plot for appendectomy time to discharge

**Fig. 4:**
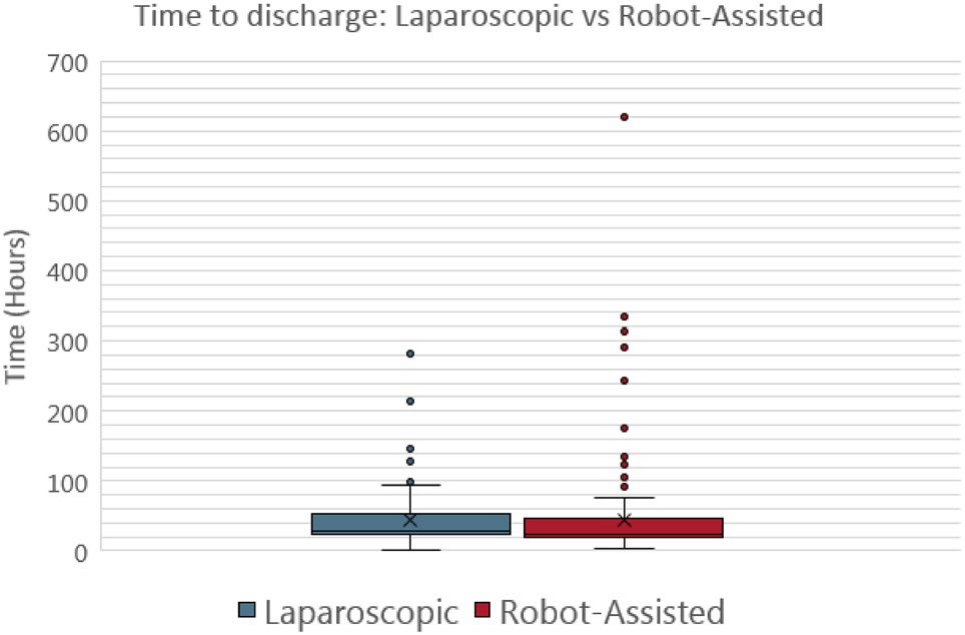
Box-and-whisker plot for Cholecystectomy time to discharge

We utilized the Tukey’s fences methodology to determine any statistical outliers, as represented by the box-and-whisker plots shown below (Figs. 1, 2, 3, 4). Any data point lying more than (1.5 × interquartile range [IQR]) below the first quartile or (1.5 × IQR) above the 3rd quartile of the data set was considered an outlier. These outliers, summarized in Table 1, were not included in the data sets analyzed.

**Table 1:** characteristics of outliers

Procedure	Statistical outliers			
	Unique	OR time	Time to discharge	Both
Laparoscopic appendectomy	13	5	11	3
Robot-assisted appendectomy	8	2	6	0
Laparoscopic cholecystectomy	10	4	7	1
Robot-assisted cholecystectomy	20	10	12	2
Appendectomy totals	21	7	17	3
Cholecystectomy totals	30	14	19	3
Laparoscopic totals	23	9	18	4
Robot-assisted totals	28	12	18	2
Grand totals	51	21	36	6

### Patient characteristics

Of the 461 total study patients, 191 underwent appendectomies, and 270 underwent cholecystectomies. Among the patients who were provided appendectomies, 110 underwent laparoscopic appendectomy and 81 underwent robotic appendectomy.

Among the patients who were provided cholecystectomies, 105 underwent laparoscopic cholecystectomy and 165 underwent robotic cholecystectomy.

For the patients receiving laparoscopic appendectomies, robotic assisted appendectomies, laparoscopic cholecystectomies, and robotic assisted cholecystectomy, the mean ages were 43.65 (range, 18–83), 44.23 (range, 20–87), 45.90 (range, 19–86), and 51.98 (range, 19–101), respectively, and 55/110 (50%), 41/81 (50%), 69/105 (65%), and 98/165 (59%) were of female sex, respectively.

### Findings for primary and secondary end points, (Table 2)

**Table 2:** Summarizes the results of the primary and secondary endpoints for each procedure

APPENDECTOMIES				
	LAP APPY	ROBO APPY	Difference	*P* value
Primary endpoints				
OR Time (mean, hours)	1.402	1.361	0.041	0.304
DC time (mean, hours)	38.26	28.349	9.911	0.010
Secondary endpoints				
Conversion to open	1/110	0/85		
Readmission	0/110	0/85		

CHOLECYSTECTOMIES				
	LAP CHOLY	ROBO CHOLY	Difference	*P* value
Primary endpoints				
OR time (mean, hours)	1.692	1.634	0.058	0.196
DC time (mean, hours)	35.95	28.46	7.49	0.002
Secondary endpoints				
Conversion to open	9/105	0/165		
Readmission	1/105	2/165		

*P* value of < 0.05 is considered statistically significant

For the appendectomies (Fig. 1), the mean intraoperative time was not significantly different between the laparoscopic and robotic-assisted procedures (1.402 vs 1.361 h; *P* value = 0.304) (Fig. 1, 2). However, the mean time to discharge between the laparoscopic and robotic-assisted procedures was statistically significant with laparoscopic procedures having a higher mean time to discharge (38.26 vs 28.349 h; *P* value of 0.010) (Fig. 3, 4).

For the cholecystectomies (Fig. 2) the mean intraoperative time was not statistically significant between the laparoscopic and robotic-assisted procedures (1.692 vs 1.634 h; *P* value = 0.196). However, the mean time to discharge between the laparoscopic and robotic-assisted procedures was statistically significant with the laparoscopic procedures having a higher mean time to discharge (35.95 vs 28.46 h; *P* value of 0.002) (Fig. 4).

### Conversion to open procedures

Of the robotic-assisted procedures, none of the patients had planned to have open procedure from the beginning, and there were no intraoperative conversions to open procedures.

For the laparoscopic procedures, one appendectomy and nine cholecystectomies were converted intraoperatively to open procedures, whereas none of the robotic-assisted surgeries were converted to open procedures (Table 3).

**Table 3:** Exclusions due to start as open and Conversions to open procedure

Laparoscopic procedures—total 225	
APPENDECTOMIES	
Planned open: 8 (EXCLUDED)	Converted to open: 1/110
CHOLECYSTECTOMIES	
Planned open: 12 (EXCLUDED)	Converted to open: 9/105

Robotic-assisted procedures—total 246	
APPENDECTOMIES	
Planned open: 0 (EXCLUDED)	Converted to open: 0/81
CHOLECYSTECTOMIES	
Planned open: 0 (EXCLUDED)	Converted to open: 0/165

As shown in Table 4, 4.65% of laparoscopic procedures were converted to open procedures intra-operatively, whereas no procedures were converted to open procedures for the robotic-assisted procedures.

**Table 4:** Describes conversions to open procedures

Procedure	Total	Planned open*	Converted to open	Converted to open (%)
Appendectomy	110	8	1	0.91
Cholecystectomy	105	12	9	8.57
Total	215	20	10	4.65

*Excluded at start

### Rate of readmission within 30 days

The rate of readmission within 30 days was evaluated for all patients by review of medical records. All readmissions were for patients who underwent cholecystectomies and none for the patients who underwent appendectomies. Out of the total 491 study participants, (461 study patients, where 20 were excluded as outliers and 10 were excluded as they were converted to open), there were five readmissions to the hospital within the first 30 days.

All readmissions were either robotic-assisted cholecystectomies (2/165), or laparoscopic cholecystectomies converted to open (1/9/105) (Table 5).

**Table 5:** Readmission rate

Variable	LAP	RA	Total
Readmission	1	2	3
No readmission	104	162	266
Total	105	164	269

No completed laparoscopic cholecystectomies were readmitted, nor laparoscopic or robotic-assisted appendectomies.

One case (Case # 2) was excluded as it was an outlier. A second case (Case # 4) was excluded, because it had started as a planned open procedure.

Case #1 underwent robotic-assisted cholecystectomy and was readmitted for small bowel obstruction 6 days after the cholecystectomy. An exploratory laparoscopic procedure for lysis of adhesions was performed.

Case #2 underwent robotic-assisted cholecystectomy and was readmitted for gastrointestinal bleeding 11 days after the cholecystectomy but was excluded for being an outlier.

Case #3 underwent a laparoscopic converted to open cholecystectomy and was readmitted for wound infection 28 days after the cholecystectomy. There was a large amount of serum with an abscess at the incision site with purulent drainage. Open drainage and lavage were performed.

Case #4 underwent an open cholecystectomy and was readmitted with small bowel obstruction 30 days after the cholecystectomy. An exploratory laparoscopic procedure was performed for lysis of the adhesions. This case was excluded for having started as an open procedure.

Case #5 underwent a robotic-assisted cholecystectomy and was readmitted for congestive heart failure and pneumonia 12 days after the cholecystectomy. This may have been post operative sequelae; however, this patient had been admitted several times within the preceding several months with a similar chief complaint, so this may have been a frequent diagnosis.

The readmission rate was analyzed using nominal data. The two groups included either patients who were readmitted to the hospital within 30 days or patients that were not readmitted to the hospital within 30 days. The Fisher’s exact test was used to determine whether or not there was a statistical difference between the readmission rates of laparoscopic and robotic-assisted procedures. The *P* value was above 1.00, indicating that the comparison was not statistically significant.

## Discussion

Our study demonstrated that surgical intraoperative time was equal for both laparoscopic and robotic-assisted approaches for the appendectomies and cholecystectomies, but post-operative time and recovery was significantly shorter for the robotic approach.

This study was unique in that two surgeons identical in skill level evaluated the operative duration of laparoscopic and robotic approaches, as well as the post-operative length of stay. Intraoperative time can be considered a surrogate of utilization of OR services, mainly special supplies and specialized OR staff. Other studies have also demonstrated better clinical outcomes for robotic-assisted procedures, including estimated blood loss and post-operative morbidities [13–15].

In our study, peri-operative care was primarily provided by hospitalists who were dedicated to patient care and were strongly encouraged to discharge patients, with surgeon approval, in an expedient fashion. This fact, along with the retrospective design of this study, allowed us to compare the two surgical approaches without surgeon bias toward either procedure type.

All robotic-assisted surgeries in this study were all performed exclusively by Surgeon B, a completely trained robotic surgeon. It is not clear whether robotic training improves laparoscopic skills; however, we made the assumption that identical surgeons in skill level would have equal laparoscopic skills. If true, this could have financial implications for credentialing of better, faster surgeons; however, this may mean an increased likelihood for a preference for laparoscopic surgery, which in turn may lead to more intraoperative conversions to open procedure.

The findings of this study were clinical, not financial, in regard to operative and post-operative time. The saving of time associated with the robotic approach may reflect better clinical outcomes such as recovery and reduced risks of complication.

In this study, the risk of conversion to open procedure was significantly higher for the laparoscopic group, and this conversion rate may reflect a more technically difficult procedure. Moreover, this may indicate that the robotic approach is a more versatile technique for handling technically difficult cases and may reduce the risk of conversion to open procedures.

One limitation of this study may have been that because more robotic cholecystectomies were performed than laparoscopic, more difficult cases may have been left to laparoscopic surgeon, increasing the risk of conversion to open procedures in this group. However, we believe that this did not affect our results, as the same surgeons and hospitals enroll less patients in the robotic appendectomy group, and both had zero conversions to open procedures. Cases that were converted to open procedures only occurred in the laparoscopic group, which may support the notion that robotic surgery is a better platform to manage more difficult surgical cases.

Another limitation is the exclusion of the outliers. If outliers were included, data and conclusions would have been influenced by a few and we would strongly assume that being an outlier most likely is the result of other complex medical conditions not related to the surgical approach that the surgical intervention modality might not have affected the overall medical condition leading to being an outlier.

The robotic surgical platform has been FDA approved for clinical use in the United States for over two decades and has been increasing in use by general surgeons in recent years. In addition, robotic appendectomies and cholecystectomies are very common and considered to require only entry-level robotic surgery skills. Moreover, many case reports have been published showing the benefits and risks of this surgical methodology; most have concluded that while visualization is improved with the binocular vision in robotic-assisted procedures, the operative time is longer and the cost per case is higher due to higher instrument costs and OR time.

To consider promoting robotic surgery as the gold standard approach, and to truly measure the cost savings of either procedure type, hospital systems must calculate global savings instead of single item savings, along with the readmission factor, the conversion factor, and the patient satisfaction and surveys and reputations.

The lower risk for conversion carried by the robotic-assisted approach is a very attractive point for patients to desire robotic surgery vs laparoscopic approach. Thus, in our opinion, it is a matter of short time before robotic surgery will become the gold standard, as clarity of evidence supporting superiority of clinical outcomes and length of stay will supersede costs of hospitalization, equipment, supplies, and specialized staff. Moreover, these costs should decrease over time.

Ultimately as physicians’ data supporting clinical and fiscal superiority for robotic surgery continue to tip the scale toward robotic surgery, hospital and healthcare systems may consider global savings including patients’ satisfactions as a tool to market to patients. Robotic surgery seems to be superior in the risk to conversion to open procedure and shorter hospital stay.

This study did not examine clinical outcomes. Future studies should examine additional clinical outcomes, such as estimated blood loss, post-operative wound infections and all surgical complications.

## Data Availability

The research data is confidential for each individual patient.
